# Emergency Endovascular Repair of a Large Ruptured Isolated Internal Iliac Artery Aneurysm in a 98-Year-Old Patient: A Case Report

**DOI:** 10.3390/jcm15218226

**Published:** 2026-10-22

**Authors:** Raon Kang, Seokjin Hong, Inae Hwang, Pa Hong, Yangwon Kim

**Affiliations:** 1 Department of Radiology, Gyeongsang National University School of Medicine and Gyeongsang National University Hospital, Jinju 52727, Republic of Korea; 2 Department of Radiology, Samsung Changwon Hospital, Sungkyunkwan University School of Medicine, Changwon 51353, Republic of Korea

**Keywords:** internal iliac artery aneurysm, aneurysm rupture, endovascular aneurysm repair, coil embolization, endoleak, case report

## Abstract

**Background/Objectives:** Isolated internal iliac artery aneurysm (IIAA) is rare and carries a high mortality when ruptured. Management is particularly challenging in very elderly, anticoagulated patients in whom open repair may carry substantial perioperative risk. Herein, we report a case that illustrates device selection when a marked common iliac–external iliac size discrepancy precludes bridging with the available iliac limbs. **Case Presentation:** A 98-year-old man receiving apixaban presented with lower abdominal pain, nausea, and diarrhea. Computed tomography demonstrated a ruptured right IIAA measuring 79 × 72 mm in the axial plane, with retroperitoneal hematoma. A marked size discrepancy between the common iliac artery (20 mm) and external iliac artery (approximately 10 mm), together with vessel tortuosity, made common iliac–external iliac bridging with an iliac limb unsuitable with the devices available. Emergency endovascular aneurysm repair with a full aortic bifurcated endograft was combined with coil embolization of the inferior gluteal artery and gelatin sponge embolization of the sac. The contralateral internal iliac artery, which harbored a 31 mm aneurysm, was preserved. A suspected type IV endoleak had resolved on CT at day 4, and the patient was discharged on postoperative day 8 without complications. At 6-month follow-up, the aneurysm sac had decreased from 79 × 72 mm to 72 × 62 mm in the axial plane with no evidence of endoleak, and he remained ambulatory without pelvic ischemic symptoms. **Conclusions:** A full aortic bifurcated endograft combined with coil and sac embolization may be a feasible bail-out option when a marked common iliac–external iliac size discrepancy precludes bridging with the available iliac limbs, even in a nonagenarian receiving apixaban.

## 1. Introduction

Isolated internal iliac artery aneurysm (IIAA) is a rare and potentially fatal condition. Because of its deep pelvic location and nonspecific symptoms, diagnosis is often delayed; in a review of 94 reported atherosclerotic cases, 40% presented with rupture and the overall mortality was 31% [1]. Several treatment options exist, including open surgical repair, coil embolization, stent-graft deployment, and combined approaches [2,3]. When the common iliac artery is much wider than the external iliac artery, a standard iliac limb may not seal both ends of a common-to-external iliac bridge. Previously reported solutions include tapered limbs delivered through an up-and-over or crossover approach [4,5], iliac limbs deployed upside down [6,7,8,9,10,11,12], customized tapered stent-grafts [13], a tapered aorto-uni-iliac converter placed in the common iliac artery [14], and aortic main-body devices [15,16]. Herein, we report a case of large ruptured IIAA in a 98-year-old patient receiving apixaban, in whom this mismatch precluded bridging with the iliac limbs available for emergency use. A full aortic bifurcated endograft was used as the definitive exclusion device, combined with coil embolization of the outflow, gelatin sponge embolization of the sac, and preservation of the contralateral internal iliac artery. The CARE checklist for this report is provided in Table S1.

## 2. Case Presentation

A 98-year-old man with a history of hypertension and peripheral arterial disease presented to a local clinic with a 1-day history of lower abdominal pain, nausea, and diarrhea. His medications were losartan and apixaban 2.5 mg. According to the referral records, apixaban had been prescribed for lower-extremity arterial insufficiency; the dosing frequency and the timing of the last dose were not documented. Anti-Xa activity was not measured, so the anticoagulant effect at the time of the procedure could not be confirmed. He was transferred to our institution after contrast-enhanced computed tomography (CT) at the referring institution demonstrated a ruptured right IIAA with retroperitoneal hematoma.

On arrival he was hemodynamically stable (blood pressure 160/100 mmHg, pulse 93 beats/min, respiratory rate 20 breaths/min, SpO_2_ 97% on room air). Laboratory studies showed a decreased hemoglobin level of 11.0 g/dL (reference range, 12.7–17.3 g/dL). The activated partial thromboplastin time was mildly prolonged at 54.4 s (reference range, 23–41 s), whereas the international normalized ratio was within normal limits at 1.18 (reference range, 0.8–1.2). Serum creatinine was normal at 0.84 mg/dL.

**Figure 1:**
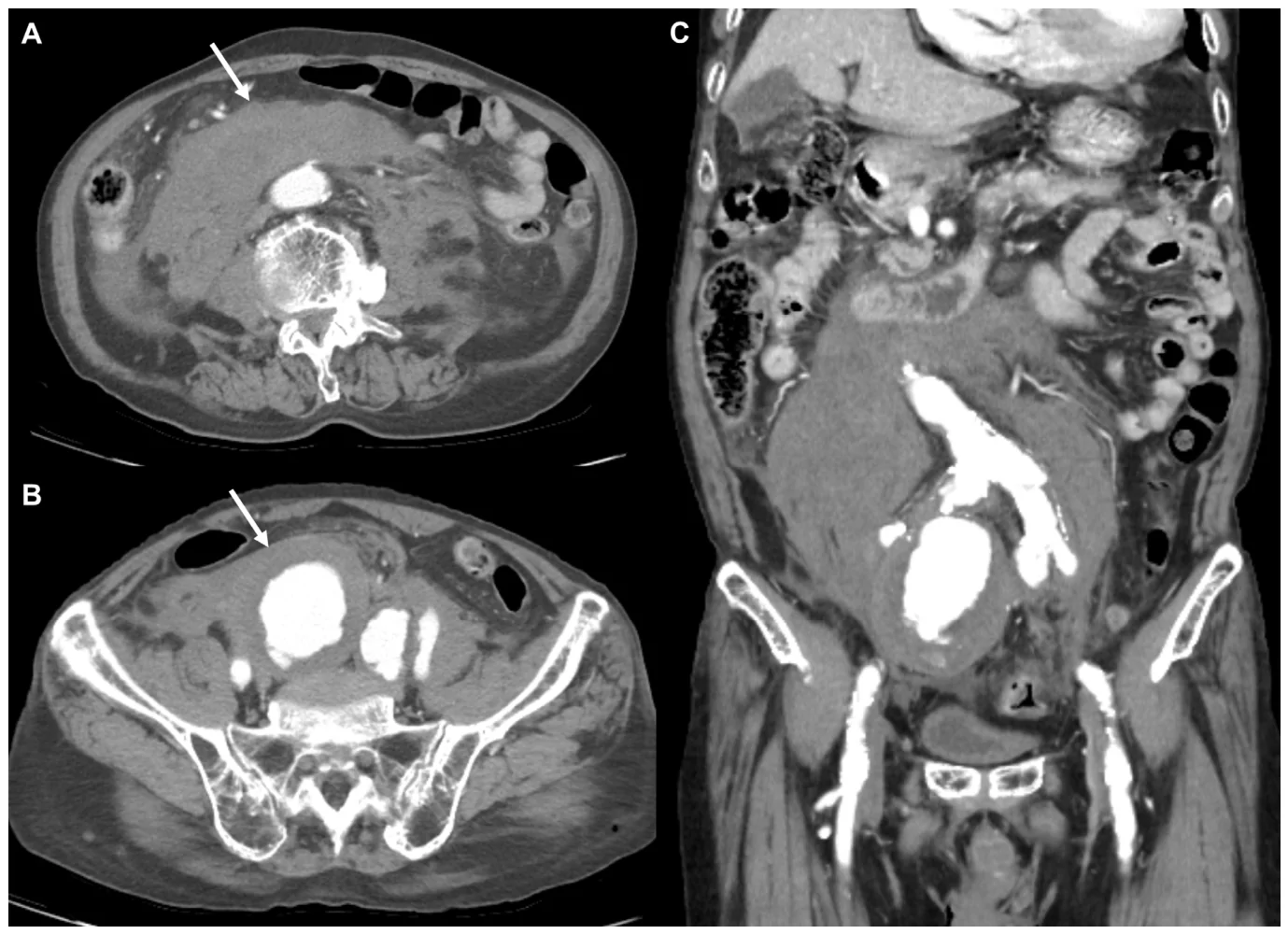
Initial CT. (**A**) Axial image demonstrates retroperitoneal hematoma (arrow). (**B**) Axial image demonstrates the ruptured right isolated internal iliac artery aneurysm (arrow). (**C**) Coronal image demonstrates the right internal iliac artery aneurysm and the surrounding retroperitoneal hematoma.

On this CT, the aneurysm measured 79 × 72 mm in the axial plane (Figure 1). The maximum diameter and the diameter perpendicular to it were measured as the outer wall-to-outer wall distance, including intraluminal thrombus, on axial images at the level of maximum diameter. The ipsilateral internal iliac artery (IIA) ostium measured 19 mm, the right common iliac artery (CIA) 20 mm, and the right external iliac artery (EIA) approximately 10 mm. The infrarenal aorta measured approximately 21 mm in diameter, with a 20 mm straight segment below the left renal artery and an infrarenal (β) angulation of approximately 56°. A 31 mm contralateral left IIAA was also present, without evidence of rupture. No concomitant abdominal aortic aneurysm was identified.

Given the aneurysm size, the rupture, and the anatomy, coil embolization alone was deemed insufficient for definitive treatment. Bridging the CIA to the EIA with an iliac limb across the IIA ostium was also unsuitable because of the marked size discrepancy between the two vessels and significant vessel tortuosity. Because of the emergent presentation, and because no specific reversal agent for apixaban was available at our institution at that time, anticoagulation reversal was not performed.

**Figure 2:**
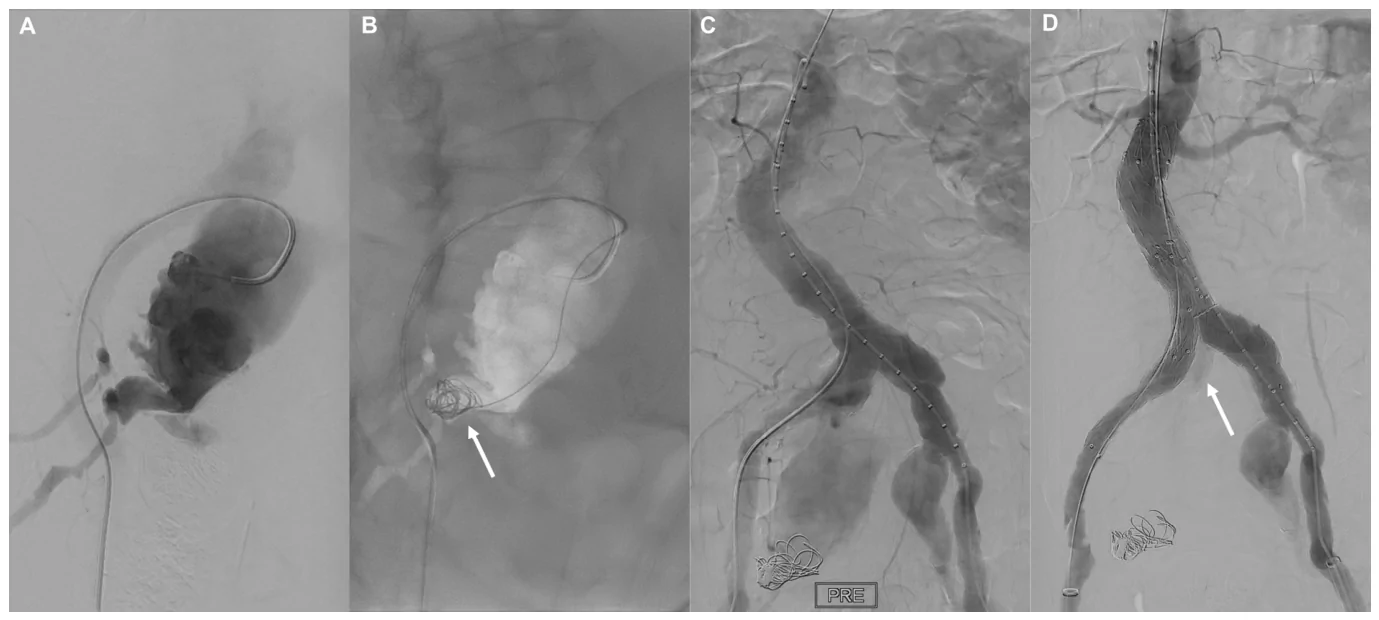
Intraoperative angiography. (**A**) Selective angiography of the right internal iliac artery demonstrates the aneurysm sac. (**B**) Coil embolization of the inferior gluteal artery ostium (arrow), followed by gelatin sponge embolization of the sac. (**C**) Subsequent aortography demonstrates bilateral internal iliac artery aneurysms. (**D**) Final angiography demonstrates successful deployment of the bifurcated endograft with a suspected type IV endoleak (arrow).

Emergency endovascular aneurysm repair (EVAR) combined with coil embolization was performed under general anesthesia. Both common femoral arteries were accessed, and heparin (3000 IU) was administered during the procedure. The right IIAA was catheterized with a 0.035-inch guidewire (Terumo, Tokyo, Japan) and a cobra catheter (Cook Medical, Bloomington, IN, USA). A microcatheter (Progreat Lambda; Terumo, Somerset, NJ, USA) was advanced to the inferior gluteal artery ostium, and coil embolization using detachable coils (10–16 mm; Concerto; Medtronic, Irvine, CA, USA, and Interlock; Boston Scientific, Marlborough, MA, USA) was performed to prevent retrograde filling of the aneurysm sac. The superior gluteal artery ostium was already near-occluded and was therefore not embolized with coils. Gelatin sponge particles (NexGel; Next Biomedical, Incheon, Republic of Korea; 1000–1600 μm and 2000–3500 μm) were then injected into the aneurysm sac. An aortic body graft (Endurant IIs; Medtronic, Santa Rosa, CA, USA; 25 × 103 × 14 mm) was then deployed in the infrarenal abdominal aorta, followed by the contralateral limb (16 × 93 × 24 mm) and the ipsilateral limb (16 × 124 × 13 mm). Device dimensions are given as proximal diameter × length × distal diameter. The left IIA was preserved to maintain pelvic perfusion, and its 31 mm aneurysm was left untreated for surveillance. Completion angiography demonstrated successful exclusion of the aneurysm with a suspected type IV endoleak (Figure 2).

**Figure 3:**
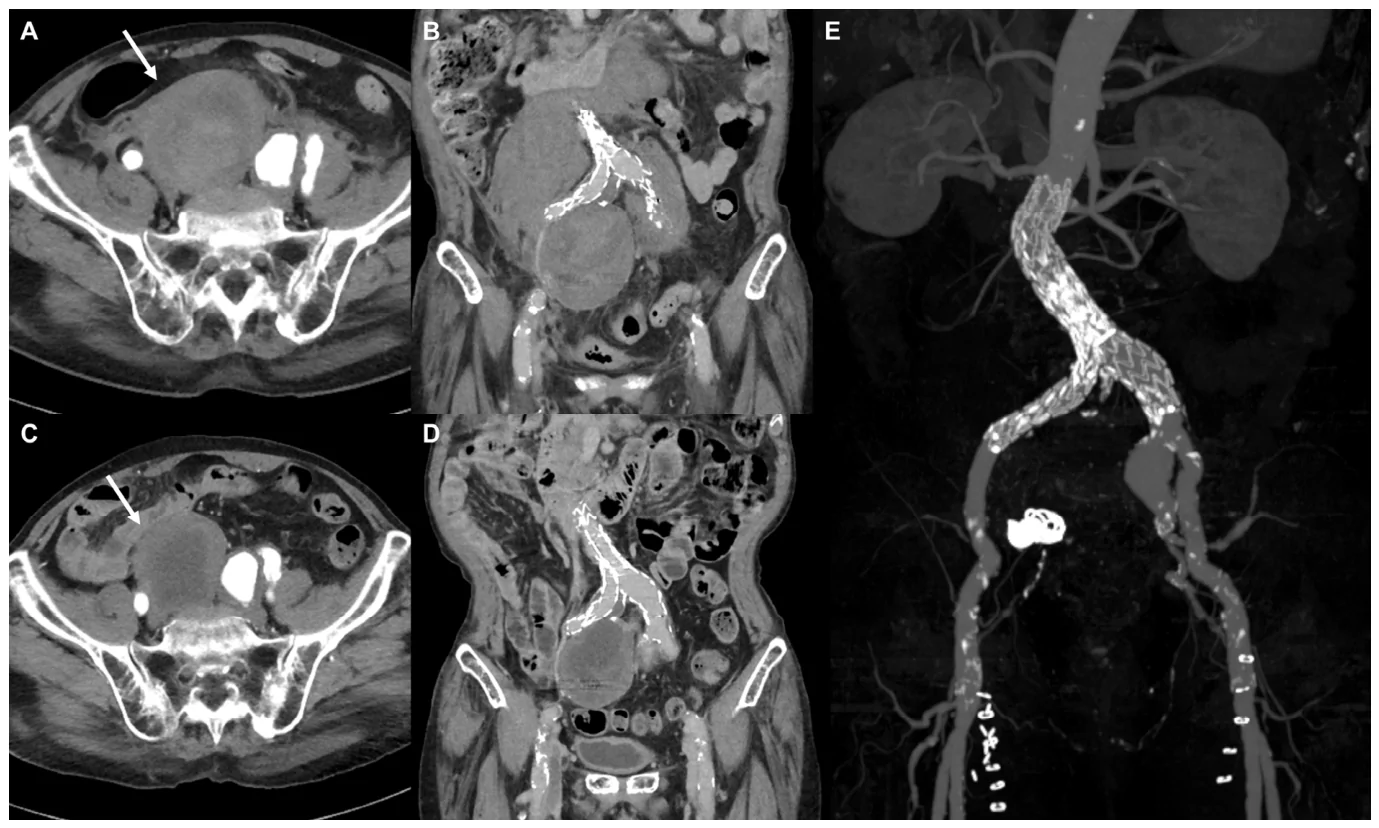
Follow-up CT. (**A**,**B**) Axial and coronal images at 4 days demonstrate complete exclusion of the right internal iliac artery aneurysm (arrow in (**A**)) and resolution of the suspected type IV endoleak. (**C**–**E**) Axial, coronal, and maximum intensity projection images at 6 months demonstrate regression of the aneurysm sac (arrow in (**C**)), with no evidence of endoleak.

One unit of packed red blood cells (250 mL) and two units of fresh frozen plasma (180 mL each) were transfused immediately after the procedure. The patient recovered without hemorrhagic or ischemic complications. Follow-up CT at 4 days confirmed complete exclusion of the right IIAA with resolution of the suspected type IV endoleak. On this CT, the sealing lengths were 38 mm in the right EIA and 32 mm in the left CIA. He was discharged on postoperative day 8. At 6-month follow-up CT, the aneurysm sac had regressed to 72 × 62 mm in the axial plane, a 7 mm decrease in the maximum diameter and a 10 mm decrease in the perpendicular diameter, with no endoleak (Figure 3). The contralateral left IIAA was stable in size. The patient remained free of buttock claudication or other pelvic ischemic symptoms and was ambulatory.

## 3. Discussion

Ruptured IIAA can be managed with open surgical repair, coil embolization, stent-graft deployment, or a combined endovascular approach [2,3]. In a single-center series of 97 patients with 125 internal iliac artery aneurysms reported by Rana et al., 15 required emergent repair, of whom four were treated endovascularly [17]. Table 1 summarizes reports of endovascular exclusion of IIAAs with covered stents, modified iliac limbs, or aortic main-body devices that were identified through a PubMed search in October 2026, supplemented by Crossref. Van Kelckhoven et al. [18] treated a ruptured IIAA by embolizing an internal iliac side branch and placing a covered stent from the common to the external iliac artery, both of which measured 16–20 mm; this tubular configuration was not feasible in our patient. When the common iliac artery is much wider than the external iliac artery, iliac limbs have been deployed upside down so that the wide end seals in the common iliac artery and the narrow end in the external iliac artery [7,9,12]. This approach has also been used in ruptured IIAAs [8,9] and in an impending rupture [10]. Tapered limbs have also been delivered through an up-and-over or crossover approach [4,5], and Kim et al. [14] placed a tapered aorto-uni-iliac converter (24 to 12 mm) within the common iliac artery for a ruptured IIAA with an adjacent common iliac artery aneurysm. Aortic main-body devices have been used to support an iliac branch device [15] and, with internal iliac coil embolization, to treat an IIAA with bilateral common iliac artery aneurysms and a normal aorta [16].

In the present case, coil embolization alone was considered insufficient given the large aneurysm size (79 mm), as complete embolization would require an excessive number of coils with uncertain efficacy. CIA–EIA bridging with an iliac limb was unsuitable with the devices available to us because of the marked size discrepancy between the CIA (20 mm) and EIA (approximately 10 mm). Only Endurant devices were available for emergency use at our institution. The available tapered configurations (16–10 mm, 16–13 mm) were undersized for the proximal CIA, whereas straight configurations (20–20 mm, 24–24 mm) would result in excessive oversizing at the distal EIA. Gemayel et al. [8] reversed an Endurant limb for a ruptured IIAA with an EIA of 12 mm. In our patient, an upside-down flared Endurant limb (16–24 mm) would have placed its 24 mm end in the CIA but its 16 mm proximal end [12] in an EIA of approximately 10 mm, an oversizing of approximately 60%. Upside-down deployment also requires off-label unsheathing and reloading of the device on the back table, which we preferred to avoid in an emergency. Significant tortuosity further argued against an iliac limb. EVAR combined with coil embolization was therefore considered the most suitable endovascular option. Given the patient’s extreme age and operative risk, an endovascular strategy was also preferred over open surgery. In the same series, endovascular repair of internal iliac artery aneurysms was associated with lower early morbidity (8% vs. 43%) and a shorter hospital stay than open repair [17].

Deploying a full aortic bifurcated endograft to exclude an isolated iliac aneurysm is a bail-out option when iliac-limited configurations are precluded by the landing-zone anatomy and the available devices, although it requires a longer device and bilateral femoral access. Tjaden et al. [9] considered a bifurcated device for a ruptured IIAA but chose an inverted limb because bifurcated repair would have taken longer in a patient in hemorrhagic shock; our patient was hemodynamically stable on arrival. Kassaian et al. [15] used a similar configuration for a leaking right IIAA: after coil embolization of the right IIA, an aortic main body was deployed in a healthy aorta and extended into the right external iliac artery. Because the contralateral IIA also harbored a 27 mm aneurysm, the main body also supported an iliac branch device that preserved left internal iliac flow. The authors considered a bifurcated endograft with surveillance of the contralateral aneurysm but did not choose it, because that aneurysm would eventually require treatment. In the present case, the contralateral IIAA measured 31 mm and was left untreated for surveillance with its native flow preserved, as discussed below. In our patient, the bifurcated endograft secured a reliable proximal seal in the infrarenal aorta and a controlled distal landing in the external iliac artery. Although use of the Endurant IIs for an isolated IIAA was outside its labeled indication, the device was deployed without modification. Among the cases in Table 1, the present case combined rupture, a bifurcated endograft as the definitive exclusion device, embolization of the outflow and the sac, and preservation of the contralateral IIA without an iliac branch device in a 98-year-old patient receiving apixaban.

The diagnosis of IIAA is frequently delayed because of its deep pelvic location and nonspecific presentation [1]. Symptoms may mimic gastrointestinal, urological, or musculoskeletal disease, or arise from mass effect on adjacent structures such as ureteric, rectal, or lumbosacral nerve compression [1]. Our patient presented with lower abdominal pain, nausea, and diarrhea, features that could readily be attributed to a gastrointestinal cause. A low threshold for cross-sectional imaging is warranted in elderly patients with unexplained lower abdominal or pelvic symptoms.

The natural history of IIAA is poorly defined, but rupture risk probably increases with size [1]. The 2024 European Society for Vascular Surgery guidelines raised the diameter at which elective repair of iliac artery aneurysms should be considered to ≥40 mm and upgraded this recommendation to Class IIa on the basis of slow growth rates and a very low risk of rupture below that diameter [19]. At 79 mm, the present aneurysm was well beyond this threshold and, in combination with the rupture, mandated emergency repair.

IIA occlusion carries a risk of pelvic ischemic complications, including buttock claudication, erectile dysfunction, and intestinal ischemia [20,21]; gluteal compartment syndrome after IIA coil embolization has also been described [22]. Preservation of internal iliac flow is associated with better outcomes. In the series by Rana et al., freedom from buttock claudication at 2 years was 25% with no IIA preserved, 68% with one, and 95% with both [17]. In patients aged over 80 years undergoing abdominal aortic aneurysm repair, sacrifice of both IIAs was associated with higher overall mortality [23]. The contralateral left IIAA measured 31 mm and was asymptomatic, below the 40 mm threshold at which elective repair should be considered [19]. Given the emergent nature of the ipsilateral repair and the risk of bilateral pelvic ischemic complications if both IIAs were occluded simultaneously, a conservative strategy was adopted for the contralateral side. This aneurysm will be managed with surveillance imaging, and intervention is reserved for significant enlargement or symptom development.

Although apixaban was not reversed before the procedure and heparin was administered during it, no hemorrhagic complication occurred. Postoperatively, the baseline thrombotic indication had to be weighed against the risk of early rebleeding from a freshly excluded sac. Apixaban was not prescribed at our outpatient clinic after discharge, and whether the patient resumed the medication elsewhere is unknown; continued imaging surveillance was therefore particularly important in this patient.

Type IV endoleak represents leakage of blood through the stent-graft fabric due to porosity in the early postoperative period; it was described mainly with first-generation devices on completion angiography in fully anticoagulated patients, is rare with newer devices, and is self-limiting [24]. The endoleak type could not be definitively established from the available completion angiogram, and the designation therefore remained presumptive. The suspected type IV endoleak resolved completely on 4-day CT without additional intervention, consistent with the expected natural history of this entity.

Durable exclusion of an IIAA requires control of both the inflow and the outflow of the aneurysm. Covering the aneurysm inflow without occluding its distal branches allows for retrograde “back-door” reperfusion of the sac through gluteal and iliolumbar collaterals. Embolization of the internal iliac circulation before endograft coverage has been recommended to prevent type II endoleak [25], and in IIAA, combined coil embolization and stent-graft placement was associated with the lowest endoleak (6%) and reintervention (6%) rates among endovascular methods [3]. Accordingly, we embolized the inferior gluteal artery and the sac before deploying the endograft. Sac embolization was intended to limit retrograde inflow from residual small branches, including the near-occluded superior gluteal artery, to aid hemostasis at the rupture site, and to accelerate sac thrombosis in a patient receiving apixaban. Gelatin sponge was used because the sac was too large to fill with coils alone. The contribution of sac embolization to the outcome cannot be separated from that of endograft exclusion and branch embolization. Incomplete collateral control can lead to a subsequent type II endoleak [25]; no endoleak was identified at follow-up in the present case.

At 6 months, the sac had regressed by 7 mm in the maximum diameter and 10 mm in the perpendicular diameter, with no identifiable endoleak. Sac regression after endovascular repair is generally regarded as a favorable prognostic sign, and these findings support maintained aneurysm exclusion through 6 months.

This case report has three limitations. As a single case, the findings cannot be generalized. The report describes a technical bail-out strategy and provides no comparative evidence on the management of IIAA. Follow-up is limited to 6 months, and longer imaging surveillance is required to confirm durable exclusion, continued sac regression, and the stability of the contralateral aneurysm.

## 4. Conclusions

The present case demonstrates that emergency EVAR combined with coil embolization can achieve technically successful exclusion of a large ruptured IIAA without periprocedural complications, even in a 98-year-old patient receiving apixaban. Careful anatomical assessment is essential for appropriate device selection, particularly when a marked CIA–EIA size discrepancy precludes bridging with the available iliac limbs. Sac regression at 6-month follow-up supports a favorable short-term outcome, and a full aortic bifurcated endograft combined with coil and sac embolization may represent a reasonable bail-out option when the landing-zone anatomy precludes an iliac-limited configuration with the available devices.

## Figures and Tables

**Table 1:** Reported endovascular exclusion of internal iliac artery aneurysms with covered stents, modified iliac limbs, or aortic main-body devices, compared with the present case.

Study	Age/Sex; Presentation	IIAA Size	CIA/EIA Diameter	Exclusion Device	Embolization	Contralateral IIA	Outcome
van Kelckhoven et al. [18]	72/M; ruptured, shock	7.5 cm	16–20 mm (no discrepancy)	Excluder iliac leg (16 to 20 mm), placed after temporary balloon occlusion	Coils in the single side branch; gelatin sponge	NR	No endoleak at 1 week; well at 2 months
Kim et al. [14]	84/M; ruptured, hypotension, on warfarin	3.1 cm (adjoining CIA aneurysm 3.3 cm)	CIA neck 19/EIA 10 mm	Zenith aorto-uni-iliac converter (24 to 12 mm) in the CIA, extended with a 12 mm iliac limb	None (hemodynamic instability)	Not treated (L IIAA 2.5 cm, unchanged)	Type II endoleak on completion angiography; complete IIAA thrombosis at 2 months
Cheong and Varcoe [4]	78/M; symptomatic (lumbosacral plexopathy)	9 cm	18/14 mm	Tapered Endurant contralateral limb, up-and-over	Microcoils in 2 anterior branches	NR	Type II endoleak on completion angiography and at 6 weeks; sac 7 cm and symptoms resolved at 9 months
Kassaian et al. [15]	68/M; symptomatic, leaking	R 46 mm; L 27 mm	R CIA 31 mm (ectatic)/EIA 8.5 mm	Zenith Flex main body; R limb to EIA; L iliac branch device	R IIA coils	Preserved with iliac branch device	Exclusion, patent pelvic arteries at 2 years
Gemayel et al. [8]	75/M; ruptured, shock	8 cm	20/12 mm	Reversed flared Endurant limb (16 to 24 mm), 24 mm end in the CIA	Coils in the distal IIA and sac; vascular plug at the IIA orifice	NR	Decompressive laparotomy; exclusion on CT at 21 days
Song et al. [7], case 1	85/M; asymptomatic	6.5 × 6.2 cm	21/11 mm	Reversed Zenith Flex leg (24 mm flare)	Vascular plug	NR	No endoleak; no follow-up CTA
Song et al. [7], case 2	82/M; ruptured	5.0 × 4.0 cm	15/11 mm	Reversed Zenith Flex leg (18 mm flare)	Coils	NR	Minimal type I endoleak; no follow-up CTA
Song et al. [7], case 3	82/M; symptomatic	4.1 cm (L IIAA 2.9 cm)	20/10 mm	Reversed Zenith Flex leg (24 mm flare)	Coils	NR	No endoleak; no follow-up CTA
Tjaden et al. [9]	88/M; ruptured, shock	11.4 cm	22.8/14.4 mm	Inverted Gore contralateral leg (27 × 16 mm)	NR	NR	Discharged after a short stay
Lacquemanne et al. [5]	72/M; ruptured, hemodynamically stable	72 mm (L)	NR	Tapered limb, femorofemoral crossover	Coils in 3 distal branches	NR	NR
Spath and Leone [10]	85/M; impending rupture	45 mm	NR	Reversed tapered Gore iliac limb (PLC181000), crossover	Vascular plugs in the IIA branches	NR	No complications; aneurysm 40 mm at 6 months
Salimi et al. [16]	63/M; asymptomatic	8 cm	Bilateral CIA aneurysms (29 and 38 mm)	EVAR (configuration NR)	IIA coils	NR	No endoleak; asymptomatic at 2 years
Kim et al. [12]	62/M; asymptomatic	R 56 mm; L 48 mm	22/11 mm	Upside-down Minos limb (14 × 24 × 80 mm)	Coils; vascular plug	L IIAA treated 1 week later with stent-grafts within the IIA	Exclusion at 1 month; transient buttock claudication
Present case	98/M; ruptured, on apixaban	79 × 72 mm (L IIAA 31 mm)	20/10 mm	Endurant IIs bifurcated endograft; ipsilateral limb to EIA	Inferior gluteal artery coils; gelatin sponge into the sac	Preserved	Sac regression without endoleak at 6 months; no pelvic ischemic symptoms

CIA, common iliac artery; CTA, computed tomography angiography; EIA, external iliac artery; EVAR, endovascular aneurysm repair; IIA, internal iliac artery; IIAA, internal iliac artery aneurysm; L, left; M, male; NR, not reported; R, right. Data for Lacquemanne et al. [5] were taken from the abstract and the article excerpt on the publisher’s website.

## Data Availability

The original contributions presented in this study are included in the article. For further inquiries, please contact the corresponding author.

## Supplementary Materials

The following supporting information can be downloaded at https://www.mdpi.com/article/10.3390/jcm15218226/s1, Table S1. CARE Checklist.

## Abbreviations

The following abbreviations are used in this manuscript:

CIA	Common iliac artery
CT	Computed tomography
EIA	External iliac artery
EVAR	Endovascular aneurysm repair
IIA	Internal iliac artery
IIAA	Isolated internal iliac artery aneurysm
