# Supplementary material for: Emergency Endovascular Repair of a Large Ruptured Isolated Internal Iliac Artery Aneurysm in a 98-Year-Old Patient: A Case Report
Source: J Clin Med. 2026 Oct 22;15(21):8226. doi: 10.3390/jcm15218226 (PMC13764070; doi:10.3390/jcm15218226)
Supplement: Supplementary file 1 [file jcm-15-08226-s001.zip › jcm-4589666-supplementary.pdf]

Table S1 CARE Checklist

Topic	Item	Checklist item description	Reported in section
**Title**	1	The words "case report" appear in the title with the diagnosis or intervention of primary focus.	Title
**Key Words**	2	Two to five key words identifying the topics of the case report, including "case report".	Keywords (six keywords, including "case report")
**Abstract**	3a	Introduction: what is unique about the case and what it adds to the literature.	Abstract (Background/Objectives)
	3b	The patient's main concerns and important clinical findings.	Abstract (Case Presentation)
	3c	The primary diagnoses, interventions, and outcomes.	Abstract (Case Presentation)
	3d	Conclusion: the main take-away lessons.	Abstract (Conclusions)
**Introduction**	4	One or two paragraphs summarizing why this case is unique (may include references).	Section 1 (Introduction)
**Patient Information**	5a	De-identified demographic and other patient information.	Section 2 (Case Presentation), first paragraph (age and sex)
	5b	Main concerns and symptoms of the patient.	Section 2, first paragraph (presenting symptoms)
	5c	Medical, family, and psychosocial history, including relevant genetic information.	Section 2, first paragraph (medical history); family, psychosocial, and genetic history not reported
	5d	Relevant past interventions and their outcomes.	No past interventions reported; current medications (losartan and apixaban) in Section 2, first paragraph
**Clinical Findings**	6	Significant physical examination and other clinical findings.	Section 2 (vital signs, laboratory findings, and CT measurements); Figure 1
**Timeline**	7	Information from this episode of care organized as a timeline.	No separate timeline figure or table; the episode of care is described in chronological order in Section 2
**Diagnostic Assessment**	8a	Diagnostic methods (e.g., physical examination, laboratory testing, imaging).	Section 2 (contrast-enhanced CT and laboratory tests); Figure 1
	8b	Diagnostic challenges.	No specific diagnostic challenges reported for this patient; the nonspecific presentation of IIAA is discussed in Section 3 (Discussion)
	8c	Diagnosis, including other diagnoses considered.	Section 2 (ruptured right IIAA with retroperitoneal hematoma); no formal differential diagnosis reported; symptoms that could be attributed to a gastrointestinal cause are discussed in Section 3
	8d	Prognostic characteristics, where applicable.	Section 2 (aneurysm size); Section 3 (size relative to the guideline threshold for repair)
**Therapeutic Intervention**	9a	Types of therapeutic intervention.	Section 2 (emergency EVAR with coil and gelatin sponge embolization); Figure 2
	9b	Administration of therapeutic intervention (e.g., dosage, strength, duration).	Section 2 (heparin dose, coil and particle sizes, endograft dimensions, and transfusion)
	9c	Changes in therapeutic intervention, with rationale.	No changes to the planned intervention were reported. Rationale for treatment and device selection: Sections 2 and 3. Reason anticoagulation was not reversed: Section 2

Topic	Item	Checklist item description	Reported in section
**Follow-up and Outcomes**	10a	Clinician- and patient-assessed outcomes.	Section 2 (recovery, discharge, and clinical status at 6 months)
	10b	Important follow-up diagnostic and other test results.	Section 2 (CT at 4 days and 6 months); Figure 3
	10c	Intervention adherence and tolerability, and how this was assessed.	Procedural adherence not applicable (single intervention); tolerability in Section 2 (no hemorrhagic or ischemic complications); anticoagulation after discharge in Section 3
	10d	Adverse and unanticipated events.	Section 2 (suspected type IV endoleak on completion angiography that resolved at 4 days; no hemorrhagic or ischemic complications); Figure 3
**Discussion**	11a	Strengths and limitations of this case report.	Strengths: anatomy-based device-selection strategy (Section 3), preservation of contralateral internal iliac perfusion (Section 3), and imaging and clinical outcomes documented through 6 months (Sections 2 and 3). Limitations: Section 3, final paragraph
	11b	Relevant medical literature, with references.	Section 3 (Discussion)
	11c	Scientific rationale for any conclusions.	Section 3 (Discussion)
	11d	Primary take-away lessons (without references) in a one-paragraph conclusion.	Section 4 (Conclusions)
**Patient Perspective**	12	The patient's perspective on the treatment received, when appropriate.	Not reported
**Informed Consent**	13	Whether the patient gave informed consent.	Informed Consent Statement (written informed consent for publication was obtained from the patient)
